# Two cases of gastric cancer with elevated serum levels of KL-6

**DOI:** 10.1186/s40792-024-01883-0

**Published:** 2024-04-09

**Authors:** Naoe Yanagisawa, Naohiko Koide, Harunari Fukai, Yoshinori Koyama, Yuu Ogihara, Maki Ohya

**Affiliations:** 1 Department of Surgery, Nagano Prefectural Kiso Hospital, 6613-4 Fukushima, Kiso, Nagano, 397-8555 Japan; 2 Department of Clinical Laboratory, Nagano Prefectural Kiso Hospital, 6613-4 Fukushima, Kiso, Nagano, 397-8555 Japan; 3 Department of Pathology, Shinshu University School of Medicine, 3-1-1 Asahi, Matsumoto, Nagano, 390-8621 Japan

**Keywords:** Gastric cancer, KL-6, Oxaliplatin, S-1, Tumor marker

## Abstract

**Background:**

The serum level of Krebs von den Lungen-6 (sKL-6) is a biomarker of interstitial pneumonia and has been reported to be elevated in patients with cancers. However, there have been few cases of gastric cancer (GC) with elevated sKL-6 that were treated by chemotherapy. We herein report two cases of GC with elevated sKL-6 that were treated with oxaliplatin plus S-1 (SOX) chemotherapy and discussed the resulting changes in sKL-6.

**Case presentation:**

The first patient was a 79-year-old woman complaining of loss of appetite. Esophagogastroduodenoscopy (EGD) showed a type-3 tumor in the gastric antrum and biopsy specimens showed adenocarcinoma. Computed tomography (CT) showed multiple liver metastases. sKL-6 was elevated to 1,292 U/ml, but a CT revealed no obvious lesions of the lungs, including interstitial pneumonia. The tumor was diagnosed as GC with liver metastases and elevated sKL-6. Respiratory function data were normal. SOX therapy using oxaliplatin and S-1 was performed. After 3 courses of SOX therapy, CT showed reductions of the liver metastases as well as the primary tumor, and sKL-6 was decreased to 201 U/ml. After the 44 courses, sKL-6 was slightly elevated. Chest CT showed interstitial pneumonia and chemotherapy was stopped. The patient is still alive without any metastasis 72 months later. The second patient was a 69-year-old woman complaining of upper abdominal pain. EGD revealed a type-3 tumor in the gastric antrum showing adenocarcinoma with HER2-positive pathology. CT showed multiple node metastases around the abdominal aorta. sKL-6 was elevated to 2,239 U/ml, but a respiratory function test showed no abnormalities, and CT of the lungs showed no obvious lesions. The tumor was diagnosed as GC with distant node metastases and elevated sKL-6. The patient received SOX therapy combined with trastuzumab. After 6 courses, the size of the primary tumor and multiple node metastases were reduced, and sKL-6 was decreased to 284 U/ml.

**Conclusions:**

These two cases suggest that sKL-6 may be important not only as an indicator of interstitial pneumonia in chemotherapeutic courses, but also as a tumor marker in GC patients with multiple metastases.

## Background

Gastric cancer (GC) is the sixth most common malignancy and the third leading cause of cancer-related deaths worldwide, with 1,089,103 cases and 768,793 deaths estimated in 2020 [1]. Chemotherapy is the standard treatment for GC with distant metastases. However, the prognosis of patients with Stage IV-GC is poor, and despite remarkable developments in chemotherapy, the median survival time is still 6–14 months [2, 3].

Krebs von den Lungen-6 (KL-6) is a sialylated carbohydrate antigen recognized as a biomarker of interstitial pneumonia [4]. KL-6 has also been reported to be elevated in the serum of patients with several malignant tumors [5]. However, there have been few case reports of GC with elevated serum levels of KL-6 (sKL-6) that were treated with chemotherapy.

Herein, we report two cases of GC with elevated sKL-6 that were treated with oxaliplatin plus S-1 (SOX) chemotherapy and discuss the resulting changes in sKL-6.

## Case presentation

### Case 1

**Fig. 1:**
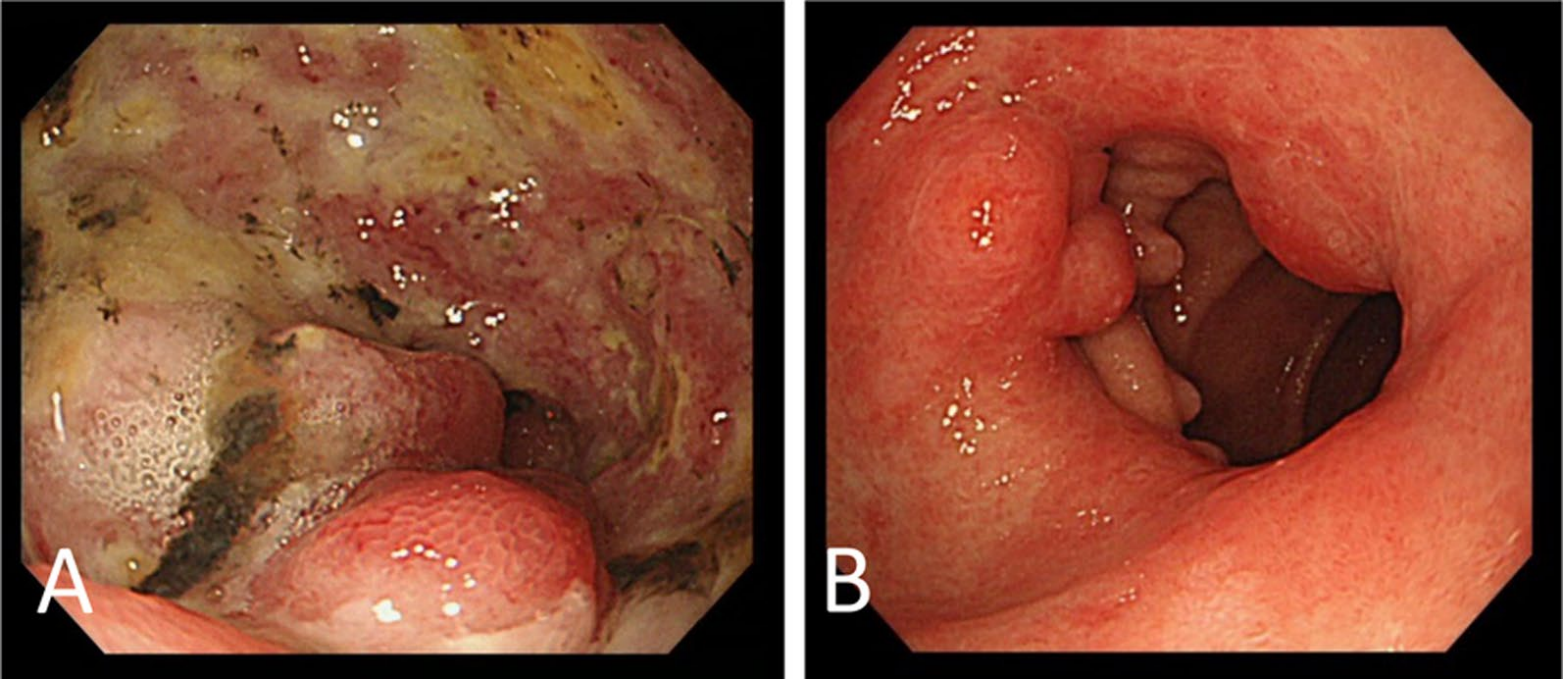
Esophagogastroduodenoscopy (EGD) in Case 1. **A** EGD shows a type-3 tumor in the gastric antrum demonstrating stenosis. **B** EGD shows significant shrinkage of the gastric tumor 66 months after starting chemotherapy

**Fig. 2:**
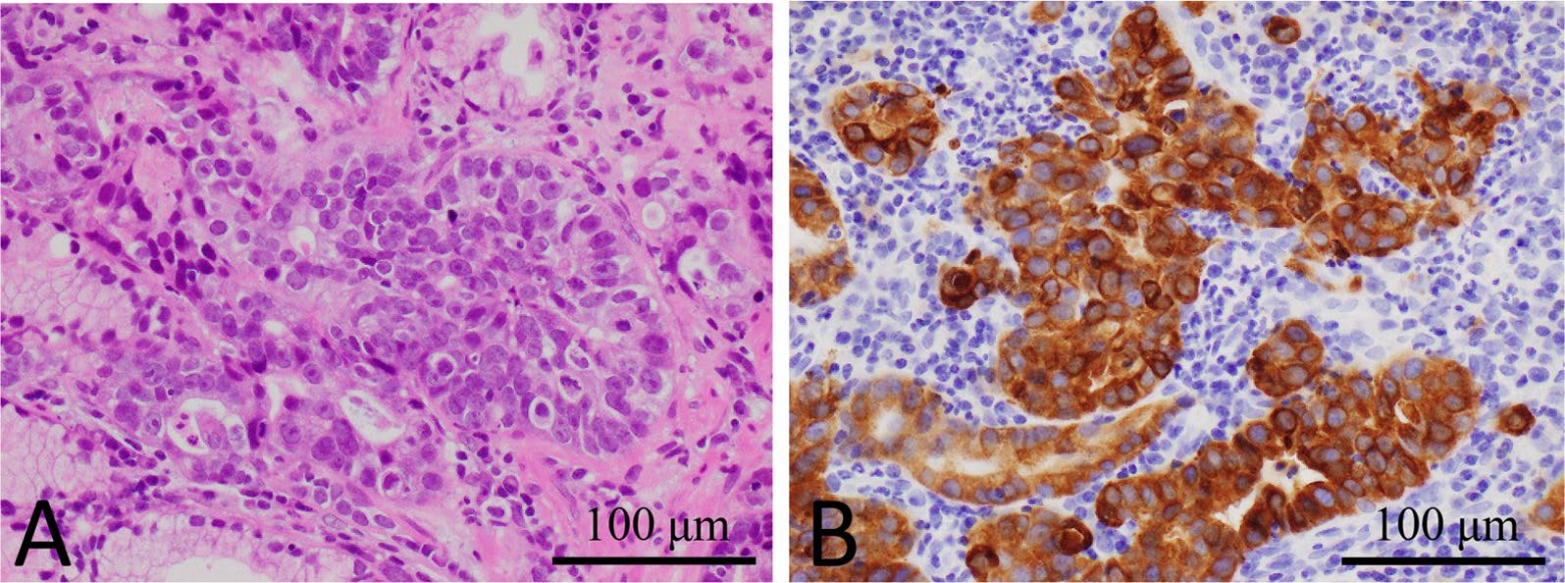
Histopathological examination of Case 1. **A** The biopsy specimens show moderately differentiated adenocarcinoma. **B** Immunohistochemically, MUC1 protein is expressed in the cytoplasm of tumor cells

**Fig. 3:**
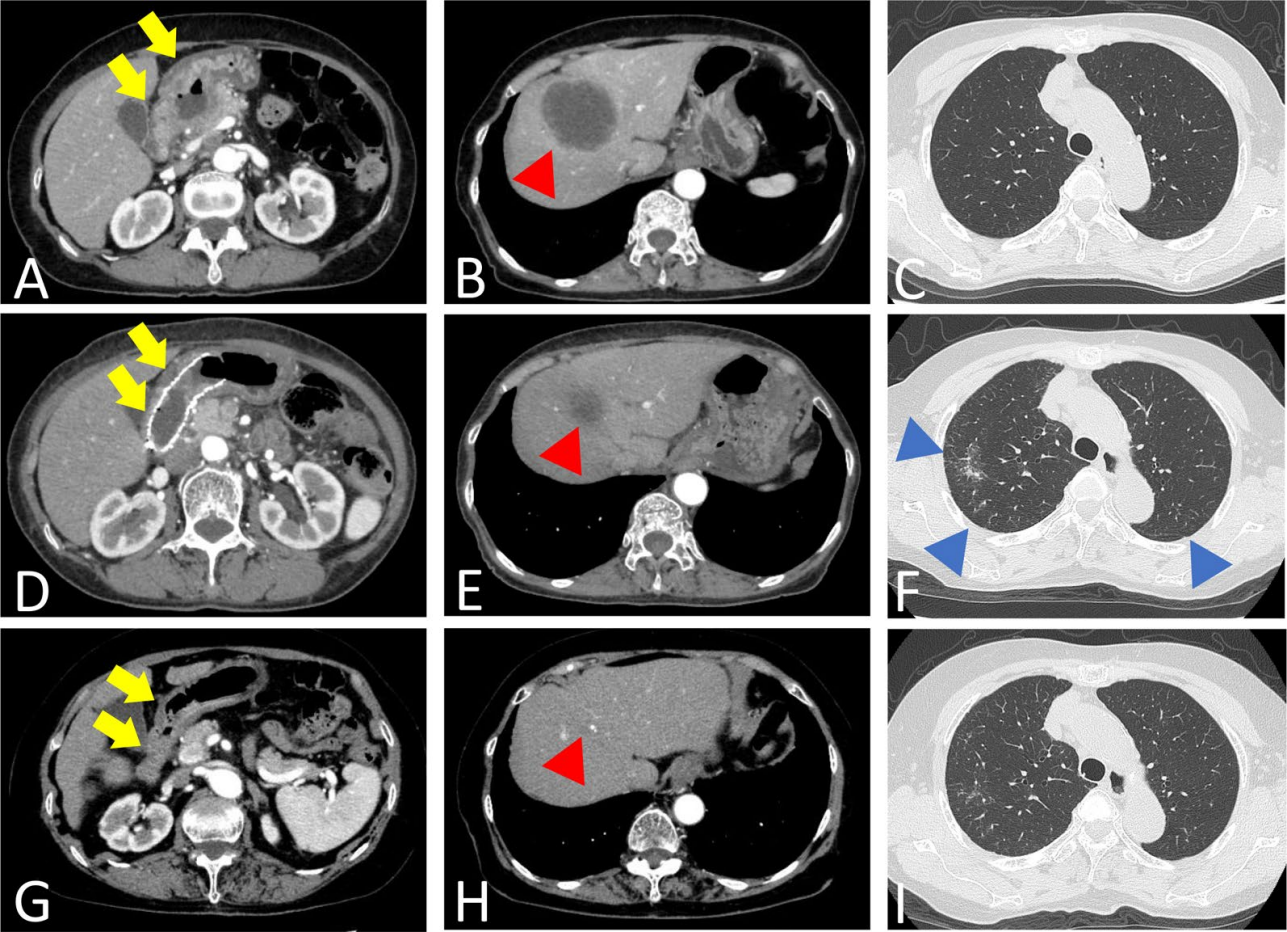
Computed tomography (CT) in Case 1. Arrows, primary gastric tumor; red arrowheads, liver metastases; blue arrowheads, lesions of interstitial pneumonia. **A** Before starting chemotherapy, the gastric tumor extends from the lower body to the pylorus of the stomach. **B** Liver metastases of up to 6.0 cm are observed. **C** CT of the lungs reveals no obvious lesions. **D** CT shows a reduction in the primary tumor after 3 cycles of SOX therapy. However, after two cycles, a metallic stent is inserted into the tumor. **E** CT shows a reduction in the liver metastases after 3 cycles of SOX therapy. **F** CT after 44 cycles of SOX therapy shows reticular and ground-glass opacities in both lung fields. **G** Sixty-six months after starting chemotherapy, CT shows significant shrinkage of the primary tumor. The metallic stent migrated and disappeared from the gastric pylorus. **H** CT shows significant shrinkage of the liver metastases. **I** Three months after stopping SOX therapy, CT shows an improvement of the interstitial lesions in both lungs

**Fig. 4:**
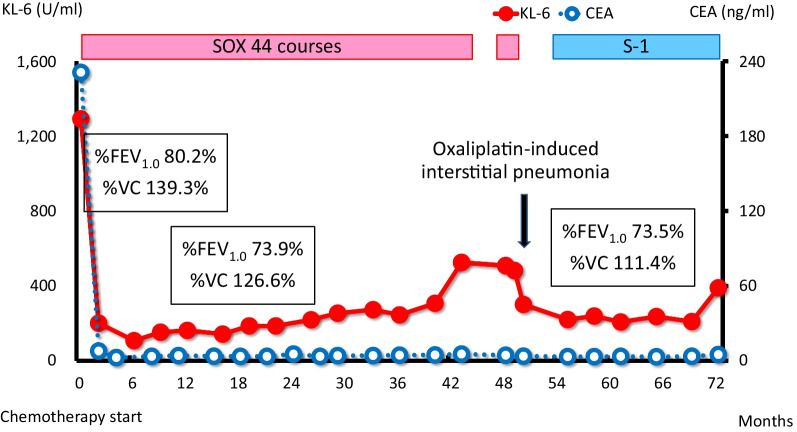
Clinical course of Case 1. KL-6, Krebs von den Lungen-6; CEA, carcinoembryonic antigen; SOX, chemotherapy using oxaliplatin plus S-1; %FEV_1.0_, percent predicted forced expiratory volume in one second; %VC, percent predicted vital capacity

A 79-year-old woman visited a clinic due to a loss of appetite and epigastric discomfort. She regularly visited the clinic for hypertension and dyslipidemia and did not smoke. She had never undergone esophagogastroduodenoscopy (EGD). For further evaluation, she visited our hospital. Physical examination revealed her weight to be 46.0 kg, her height to be 152.0 cm, and her body mass index to be 19.9 kg/m^2^. She had lost 9 kg of weight over the last 3 months. Her abdomen was soft, but her liver was palpable, 4 cm at the right hypochondrium. EGD showed a type-3 tumor located in the gastric antrum that showed severe stenosis, and an endoscope could not pass through it (Fig. 1A). Biopsy specimens taken from the tumor indicated a moderately differentiated adenocarcinoma (Fig. 2A). Immunohistochemical analyses found that human epidermal growth factor receptor type2 protein (HER2) was not expressed in the tumor cells. CT revealed that the gastric tumor extended from the lower body to the pylorus of the stomach, with regional lymph node metastases (Fig. 3A) and multiple liver metastases of up to 6.0 cm (Fig. 3B). Serum levels of carcinoembryonic antigen (CEA) were 231.0 ng/ml (normal range < 5.0 ng/ml). sKL-6 was also elevated to 1292 U/ml (normal range < 500 U/ml), but CT of the lungs revealed no obvious lesions (Fig. 3C). There was no history of medication that could cause drug-induced pneumonia. Levels of other tumor markers such as carbohydrate antigen 19–9 (CA19-9) and alpha-fetoprotein were normal. The tumor was diagnosed as GC with liver metastases and elevated sKL-6. Immunohistochemical analyses showed that MUC1 protein was expressed in the cytoplasm of the tumor cells (Fig. 2B). For respiratory function, the percent predicted forced expiratory volume in one second (%FEV_1.0_) was 80.2% and the percent predicted vital capacity (%VC) was 139.3%. The patient underwent SOX therapy combined with S-1 and oxaliplatin. S-1 was given orally for the first 2 weeks of a 3-week cycle at 100 mg/day, and oxaliplatin was infused at 80 mg/m^2^ on day 1. The dose of oxaliplatin was reduced to 80% considering her age. Due to the occurrence of pyloric stenosis after the start of the second cycle of SOX therapy, a metallic pyloric stent was inserted endoscopically. CT showed a reduction in the primary tumor (Fig. 3D) as well as the liver metastases (Fig. 3E) after 3 cycles of SOX therapy. Serum levels of CEA and sKL-6 dropped dramatically after 3 cycles of SOX therapy to 7.8 ng/ml and 201 U/ml, respectively (Fig. 4, Table 1). The efficacy of the treatment was assessed to be a partial response (PR). Subsequently, the patient received SOX therapy for a further 41 cycles. Although we sometimes delayed the drug administration of the next cycle due to bone marrow suppression, there were no severe adverse effects. Oxaliplatin-induced peripheral neuropathy was controlled using pregabalin. The PR efficacy continued over the clinical course. After 42 cycles of SOX therapy, sKL-6 increased to 525 U/ml. The patient showed no symptoms of breathlessness, cough, fever, or hypoxemia; however, CT after 44 cycles of SOX therapy showed reticular and ground-glass opacities in both lung fields, leading to a diagnosis of interstitial pneumonia (Fig. 3F). Chemotherapy was stopped promptly due to suspected oxaliplatin-induced interstitial pneumonia. Three months later, CT showed an improvement of the interstitial lesions in both lungs (Fig. 3I). Chemotherapy was resumed with only S-1 given orally. Sixty-six months after starting chemotherapy, EGD showed significant shrinkage of the gastric tumor (Fig. 1B), and no cancer cells were detected in the biopsy specimens. CT showed significant shrinkage of the primary and metastatic lesions (Fig. 3G and H). The treatment efficacy was evaluated as a complete response, and the patient is still alive 72 months after starting chemotherapy.

**Table 1:** Laboratory variations in Case 1

	Before chemotherapy	After 3 cycles of SOX therapy	Just before a diagnosis of interstitial pneumonia	Just before a restarting of chemotherapy with only S-1
Months	0	2	49	55
CEA (ng/ml)	231.0	7.8	5.4	3.3
KL-6 (U/ml)	1292	201	525	220

CEA: carcinoembryonic antigen; KL-6: Krebs von den Lungen-6; SOX: chemotherapy using oxaliplatin plus S-1

### Case 2

**Fig. 5:**
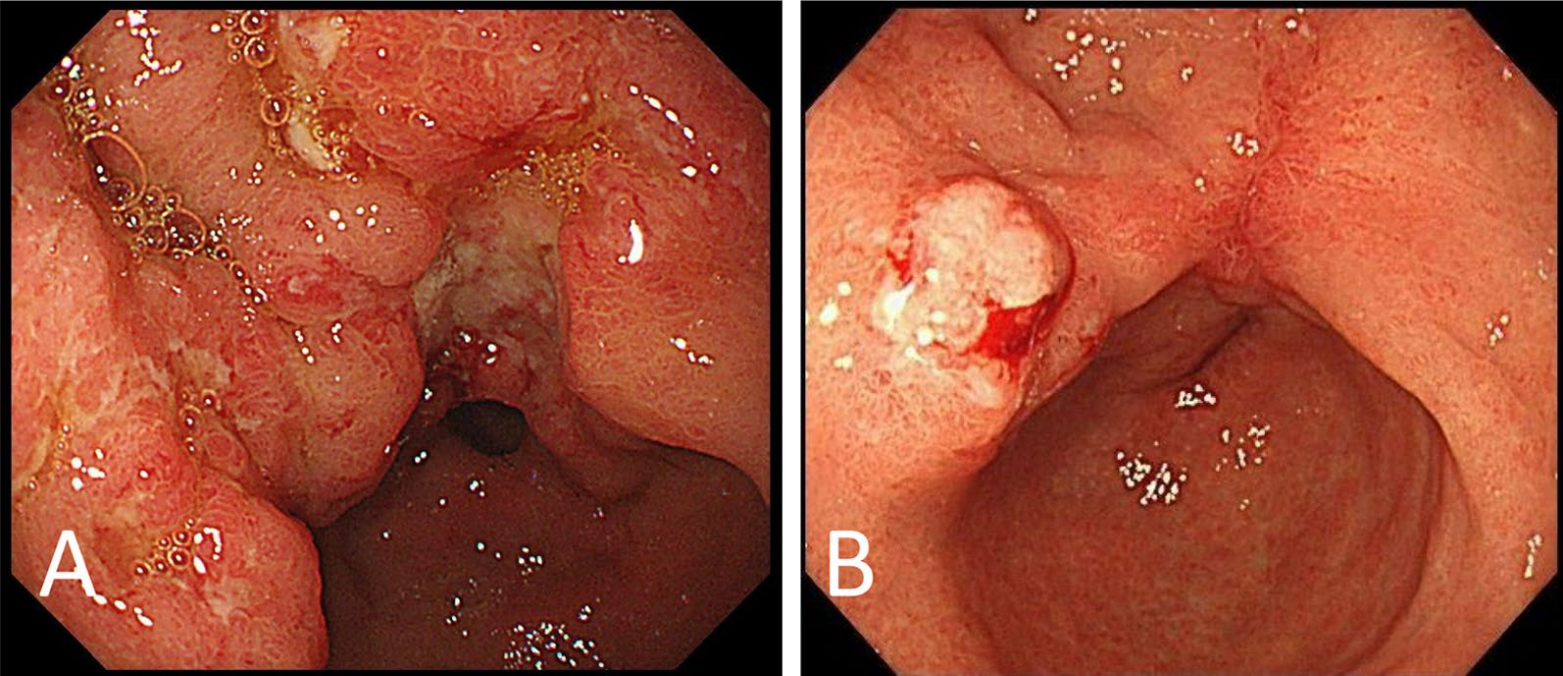
EGD in Case 2. **A** EGD shows a type-3 tumor in the gastric antrum. **B** After 3 cycles of chemotherapy, EGD shows a reduction in the size of the primary tumor

**Fig. 6:**
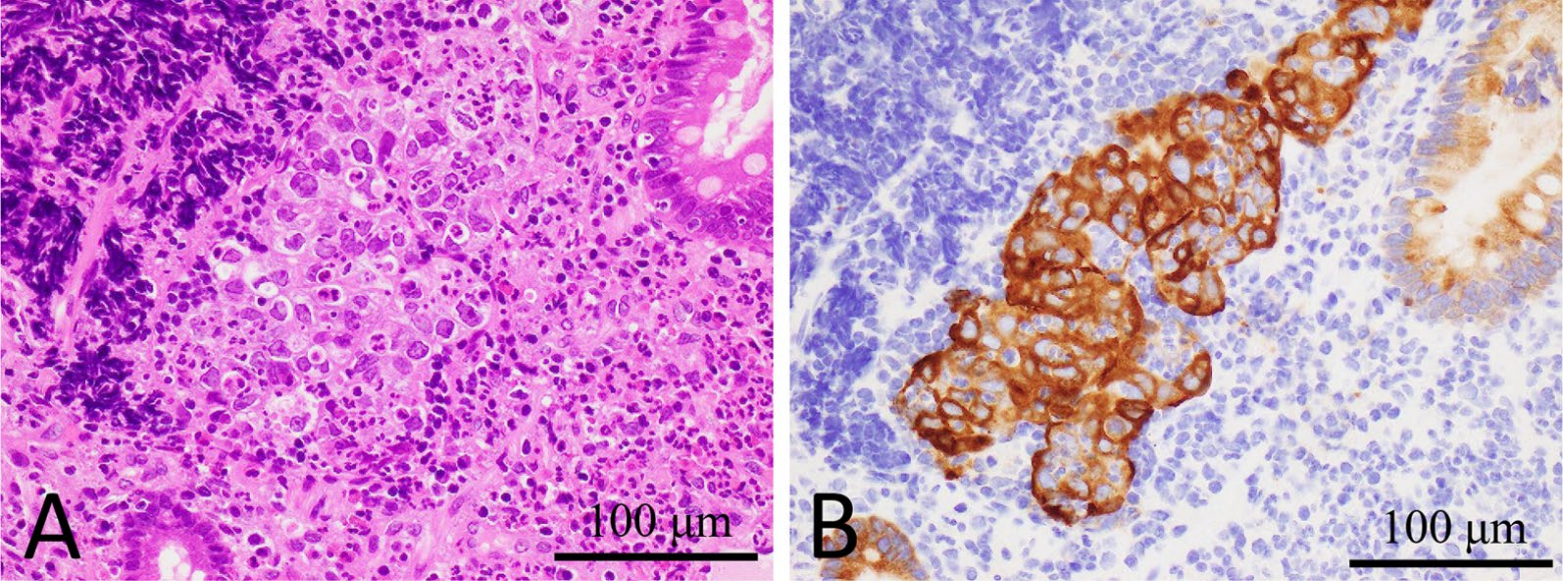
Histopathological examination of Case 2. **A** The biopsy specimens show moderately differentiated adenocarcinoma. **B** Immunohistochemically, MUC1 protein is expressed in the cytoplasm of tumor cells

**Fig. 7:**
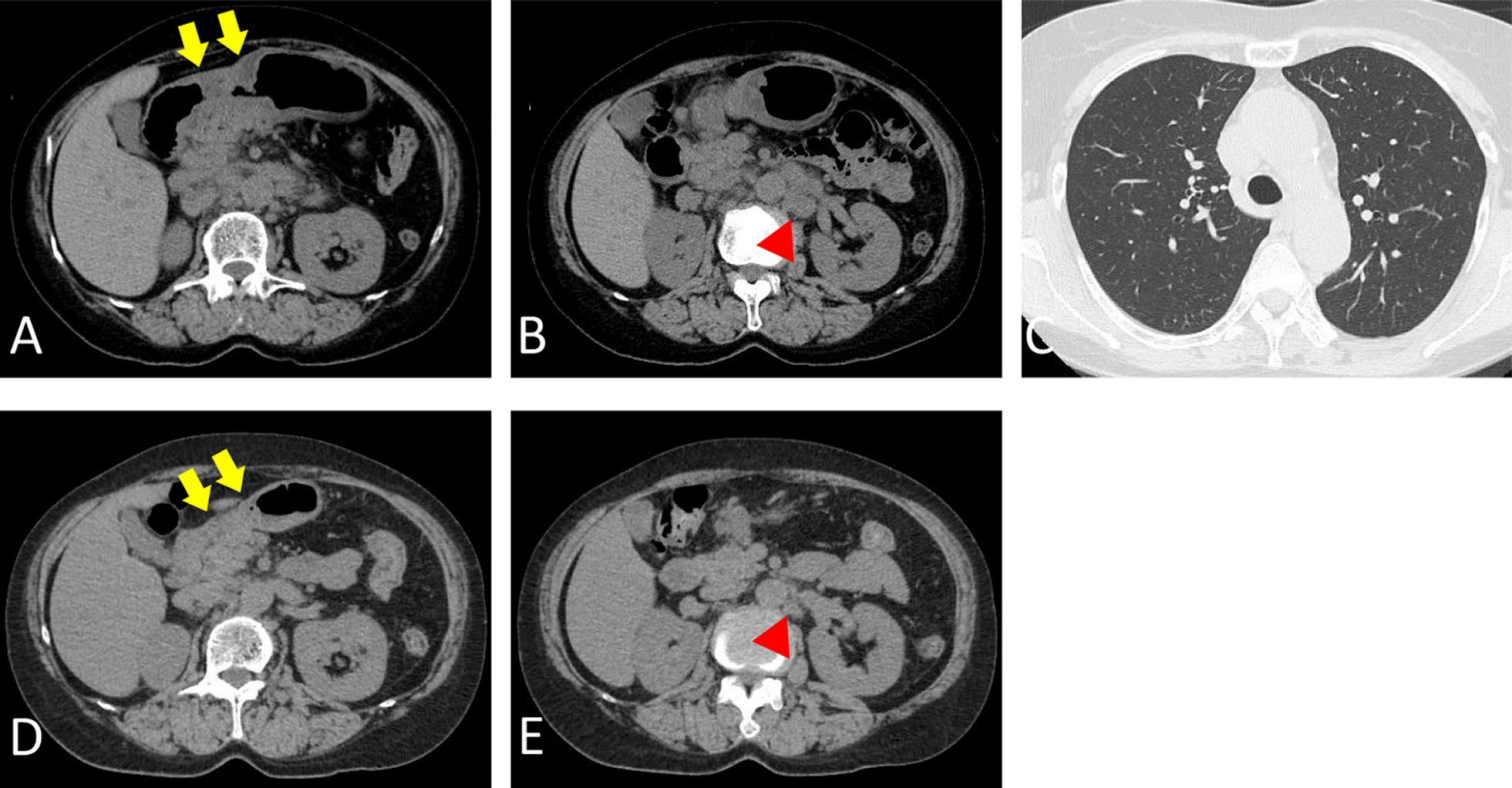
CT in Case 2. Arrows, primary gastric tumor; arrowheads, node metastases around abdominal aorta. **A** Before starting chemotherapy, CT shows a mass formed by the tumor of the gastric antrum. **B** CT shows multiple node metastases around the abdominal aorta. **C** CT of the lungs revealed no obvious lesions. **D** After 3 cycles of chemotherapy, CT shows shrinkage of the primary tumor. **E** CT shows shrinkage of the node metastases around the abdominal aorta

**Fig. 8:**
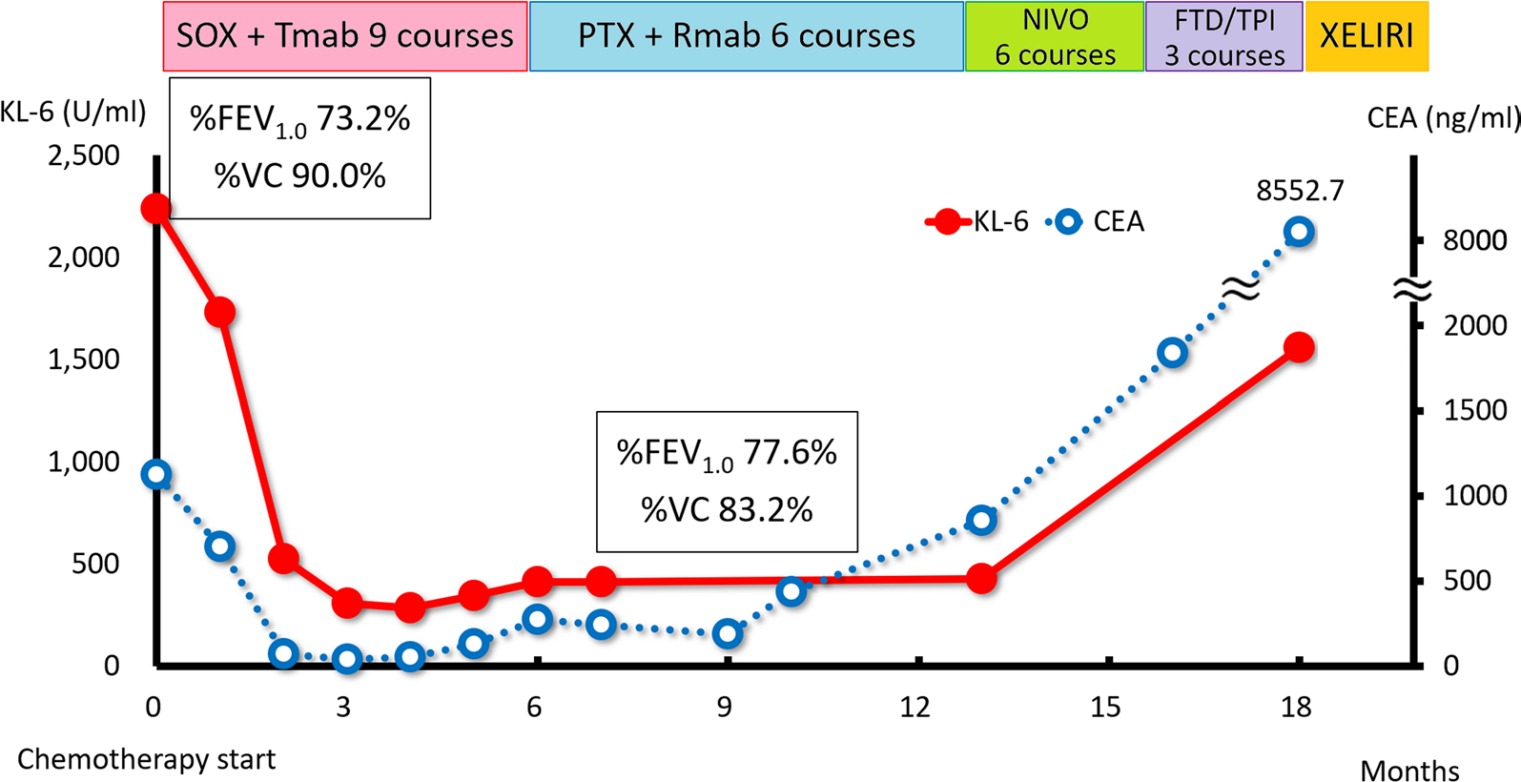
Clinical course of Case 2. KL-6, Krebs von den Lungen-6; CEA, carcinoembryonic antigen; SOX, chemotherapy using oxaliplatin plus S-1; Tmab: trastuzumab; PTX: paclitaxel; Rmab: ramucirumab; NIVO: nivolumab; FTD/TPI: trifluridine/tipiracil hydrochloride; XELIRI: capecitabine plus irinotecan; %FEV_1.0_: percent predicted forced expiratory volume in one second; %VC: percent predicted vital capacity

The second patient was a 69-year-old woman complaining of upper abdominal pain. She regularly visited the clinic for her bronchial asthma. EGD revealed a type-3 tumor in the gastric body (Fig. 5A) showing adenocarcinoma (Fig. 6A) with HER2-positive pathology. CT confirmed a tumor of the gastric body (Fig. 7A) and showed multiple node metastases around the abdominal aorta (Fig. 7B). Physical examination revealed her weight to be 58.7 kg, her height to be 159.6 cm, and her body mass index to be 23.0 kg/m^2^. Her abdomen was soft without tenderness or masses. Serum levels of CEA and CA19-9 were 1127.8 ng/ml and 24 U/ml, respectively. sKL-6 was elevated to 2,239 U/ml, but respiratory function tests showed no abnormalities (%FEV_1.0_ and %VC, 73.2% and 90.0%, respectively), and CT of the lungs showed no obvious lesions (Fig. 7C). There was no history of medication that could cause drug-induced pneumonia. The tumor was diagnosed as GC with distant node metastases and elevated sKL-6. In addition, immunohistochemical analysis showed that MUC1 protein was expressed in the cytoplasm of tumor cells (Fig. 6B). The patient received SOX therapy with trastuzumab. SOX therapy was performed in the same manner as for Patient 1, and trastuzumab was infused on day 1 (8 mg/kg for the first cycle and 6 mg/kg for subsequent cycles). After 3 cycles of chemotherapy, EGD showed a reduction in the size of the primary tumor (Fig. 5B), and CT showed reduction not only in the primary tumor (Fig. 7D), but also in the node metastases around the aorta (Fig. 7E). Furthermore, serum levels of CEA and sKL-6 were decreased to 54.0 ng/ml and 284 U/ml, respectively (Fig. 8, Table 2). The efficacy of the treatment was assessed to be PR. As serum levels of CEA were elevated after 9 cycles of SOX therapy, 2nd line chemotherapy using paclitaxel combined with ramucirumab was performed. Subsequently, the patient was treated by 6 cycles of nivolumab-therapy as the 3rd line chemotherapy and 3 cycles of trifluridine/tipiracil hydrochloride (FTD/TPI) as the 4th line chemotherapy because of new lung and liver metastases. Serum levels of CEA and sKL-6 were elevated 18 months after starting chemotherapy in the 4th line chemotherapy using FTD/TPI. The patient is alive with distant node metastases, but without hepatic nor lung metastasis, and her respiratory function data are normal.

**Table 2:** Laboratory variations in Case 2

	Before chemotherapy	After 3 cycles of 1st line chemotherapy (SOX + Tmab)	Just before 2nd line chemotherapy (PTX + Rmab)	Just before 3rd line chemotherapy (NIVO)	Just before 4th line chemotherapy (FTD/TPI)	Just before 5th line chemotherapy (XELIRI)
Months	0	2	6	13	16	18
CEA (ng/ml)	1127.8	54.0	273.1	858.5	1840.3	8552.7
KL-6 (U/ml)	2239	284	413	427	no data	1560

CEA: carcinoembryonic antigen; KL-6: Krebs von den Lungen-6; SOX: chemotherapy using oxaliplatin plus S-1; Tmab: trastuzumab; PTX: paclitaxel; Rmab: ramucirumab; NIVO: nivolumab; FTD/TPI: trifluridine/tipiracil hydrochloride; XELIRI: capecitabine plus irinotecan

## Discussion

KL-6 is a sialylated carbohydrate antigen discovered by Kohno et al. [5]. It is widely known as a biomarker of interstitial pneumonia [4]. Clinicians use sKL-6 for diagnosis of interstitial pneumonia as well as for evaluating the activity of the illness. KL-6 has also been used as a marker of tumors, and has been found to be elevated in patients with several malignant tumors. One study reported sKL-6 to be increased in 52% (17/33) of patients with lung adenocarcinoma, 18% (4/22) with lung squamous cell carcinoma, 8% (1/13) with hepatocellular carcinoma, 44% (4/9) with pancreatic cancer, and 40% (8/20) with breast cancer [5]. Ogawa et al. [6] similarly reported that sKL-6 was increased in 31% of patients with breast cancer. Kawata et al. [7] reported a case of KL-6-producing invasive thymoma, and Mogami et al. [8] reported two cases of ovarian carcinoma with elevated sKL-6. Fukuhara et al. [9] reported a case of colon cancer with elevated sKL-6, and Tsuchie et al. [10] reported a case of ovarian cancer with elevated sKL-6 and a case of colon cancer with elevated sKL-6. Finally, Yonenaga et al. [11] reported a case of gallbladder cancer with elevated sKL-6. However, to the best of our knowledge, there have been no previous reports of GC with elevated sKL-6. Indeed, although Kohno et al. [5] investigated sKL-6 levels in 19 patients with GC, no significant change was found.

In the present cases, despite the absence of interstitial pneumonia, sKL-6 was found to be elevated at GC diagnosis and was reduced following effective chemotherapy. There was no history of medications that could cause drug-induced pneumonia, like anti-cancer drugs at previous clinics. We suspected these cancer cells might produce KL-6. Therefore, it was considered that the production of KL-6 in tumor cells should be measured. However, unfortunately, we were unable to precisely detect KL-6 in tumor cells due to technical problems. KL-6 is defined as a kind of MUC1. We performed immunohistochemical staining for MUC1 in tumor tissues taken by endoscopic biopsy. Tumor cells showed a cytoplasmic positive reaction for MUC1 in both cases. There have been reported to be several patterns for the immunohistochemical localization of MUC1 and/or KL-6 [9, 11–13]. The relationship between elevated sKL-6 and staining patterns of KL-6/MUC1 has not yet been established and is still controversial. Fukuhara et al. [9] reported that sKL-6 was elevated in one colon cancer patient, with positive staining for KL-6 in the cytoplasm of cancer cells, but not in another colon cancer patient, with KL-6-positive staining found only in the apical membrane. Tanaka et al. [13] performed immunohistochemical analysis of KL-6 expression in 103 surgically resected non-small cell lung cancer (NSCLC) tissues. They reported that KL-6 expression was observed at apical membranes, circumferential membranes, and/or cytoplasm in all 103 NSCLC tissues, and that circumferential membrane and/or cytoplasmic KL-6 expression patterns were associated with a high level of sKL-6 [13]. The circumferential and/or cytoplasmic expression of KL-6 was reported to be related to malignancy when compared to expression on the apical surface in several cancers. Tang et al. [14] performed immunohistochemical analysis of ampullary cancer tissues using a KL-6 murine monoclonal antibody and found positive staining in 68.4% of all cases. They also reported that positive KL-6 expression was related to lymph node metastasis and that the prognosis of patients positive for KL-6 mucin expression was significantly poorer than those without KL-6 mucin expression [14]. According to the immunohistochemical analysis of 82 colorectal adenocarcinoma patients by Guo et al. [12], the five-year survival rate, the presence of lymph node metastasis, and the presence of liver metastasis in the patients showing positive staining in the circumferential membrane and/or cytoplasm was significantly worse when compared to that of those that showed positive staining in the apical membrane or no staining at all. Tanaka et al. [13] reported that circumferential membrane and/or cytoplasmic KL-6 expression patterns were associated with a poor prognosis in NSCLC patients who underwent curative surgery as well as a high level of sKL-6. MUC1 was predominantly present on the apical surface of normal glandular epithelial cells in many secretory organs [15, 16]. So, it is thought that circumferential membrane and/or cytoplasmic expression of KL-6 represents aberrant subcellular expression. It has been proposed that aberrant expression of MUC1 facilitates detachment of tumor cells from the primary tumor because MUC1 mediates anti-adhesion activity by interfering with cell-to-cell and/or cell-to-extracellular matrix interactions [17–22]. Guo et al. [12] and Tanaka et al. [13] suggested that aberrant subcellular expression of KL-6 might facilitate detachment of tumor cells from the primary growth in colorectal adenocarcinoma and in NSCLC, resulting in an increased ability of tumor cells to metastasize. Tanaka et al. [13] also demonstrated that aberrant expression patterns of KL-6 were associated with elevated sKL-6. In the present two cases of GC accompanied by multiple distant metastases, we demonstrated elevated sKL-6 and aberrant expression patterns of MUC1. Thus, tumors with elevated sKL-6 may tend to be accompanied by multiple metastases, like in the present two cases.

As KL-6 is mainly expressed on type 2 alveolar pneumocytes and respiratory bronchiolar epithelial cells, sKL-6 is useful as an indicator of disease activity in interstitial pneumonia [4]. It is well known that chemotherapy for metastatic cancer patients occasionally induces drug-induced interstitial pneumonia. We routinely examined sKL-6 every 3 months and performed pulmonary function tests and CT of the chest before and during chemotherapy in these patients. Although, in Case 1, sKL-6 was within the normal range during chemotherapy, it was significantly elevated after the 42 cycles of SOX therapy. We continued SOX therapy expecting CR efficacy of the tumor because no severe adverse events, including bone marrow suppression and peripheral neuropathy, were observed. We detected interstitial changes in both the lungs without any respiratory symptoms in Case 1. It is necessary that sKL-6 be periodically examined in metastatic GC patients before and during chemotherapy in order for the early detection of interstitial pneumonia.

In these cases, serum levels of KL-6 and CEA showed a similar clinical course transition. When CT scans showed a reduction in lesion sizes due to chemotherapy, serum levels of both CEA and KL-6 decreased similarly. This suggested that these cancer cells might be producing KL-6. However, in Case 1, sKL-6 increased after the 42 cycles of SOX therapy despite a decrease in serum CEA levels. It was thought that this increase in sKL-6 was not due to KL-6 production from the tumors but from the lungs.

Conversion surgery after chemotherapy has been focused as a promising tool for improving prognosis in GC patients with stage IV. Conversion surgery with R0 resection for stage IV GC could be a new therapeutic strategy to improve patient survival [23]. However, there is only one ongoing randomized controlled trial comparing survival after chemotherapy for stage IV GC with conversion surgery or continued chemotherapy [24]. In Case 1, the efficacy of chemotherapy was so good that the size of the lesions decreased well and there were no new lesions. However, she did not undergo conversion surgery due to her age. In Case 2, she was diagnosed with stage IV GC for the distant lymph node metastases. For locally advanced GC with extensive lymph node metastasis, chemotherapy followed by surgery including para-aortic lymph nodes dissection was effective for some patients [25]. However, she did not undergo conversion surgery because the lymph node metastases were widespread, extending around the common iliac arteries.

To the best of our knowledge, there have been no previous reports of metastatic GC patients with elevated sKL-6 being treated by chemotherapy. Therefore, our two patients are exceedingly rare cases.

## Conclusion

We reported 2 cases of GC in which elevated sKL-6 was observed. These two cases suggest that sKL-6 may be important not only as an indicator of interstitial pneumonia on chemotherapeutic courses, but also as a tumor marker in GC patients with distant metastases.

## Data Availability

Not applicable.

## Abbreviations

sKL-6: Serum level of Krebs von den Lungen-6
GC: Gastric cancer
SOX: Oxaliplatin plus S-1
EGD: Esophagogastroduodenoscopy
CT: Computed tomography
KL-6: Krebs von den Lungen-6
HER2: Human epidermal growth factor receptor type2 protein
CEA: Carcinoembryonic antigen
CA19-9: Carbohydrate antigen 19-9
%FEV_1.0_: Percent predicted forced expiratory volume in one second
%VC: Percent predicted vital capacity
PR: Partial response
NSCLC: Non-small cell lung cancer

## References

[1] Sung H, Ferlay J, Siegel RL (2021). Global cancer statistics 2020: GLOBOCAN estimates of incidence and mortality worldwide for 36 cancers in 185 countries. CA Cancer J Clin.

[2] Ohtsu A, Shimada Y, Shirao K (2003). Randomized phase III trial of fluorouracil alone versus fluorouracil plus cisplatin versus uracil and tegafur plus mitomycin in patients with unresectable, advanced gastric cancer: The Japan Clinical Oncology Group Study (JCOG9205). J Clin Oncol.

[3] Bang YJ, Van Cutsem E, Feyereislova A (2010). Trastuzumab in combination with chemotherapy versus chemotherapy alone for treatment of HER2-positive advanced gastric or gastro-esophageal junction cancer (ToGA): a phase 3, open-label, randomized controlled trial. Lancet.

[4] Kohno N, Kyoizumi S, Awaya Y (1989). New serum indicator of interstitial pneumonia activity. Sialylated carbohydrate antigen KL-6. Chest.

[5] Kohno N, Akiyama M, Kyoizumi S (1988). Detection of soluble tumor-associated antigens in sere and effusions using novel monoclonal antibodies, KL-3 and KL-6, against lung adenocarcinoma. Jpn J Clin Oncol.

[6] Ogawa Y, Ishikawa T, Ikeda K (2000). Evaluation of serum KL-6, a mucin-like glycoprotein, as a tumor marker for breast cancer. Clin Cancer Res.

[7] Kawata T, Tsukagoshi H, Mashimo T (2002). KL-6 producing invasive thymoma. Intern Med.

[8] Mogami T, Saji H, Yokota N (2012). Serum KL-6 for diagnosis of ovarian carcinoma associated with dermatomyositis: two case reports and characteristic clinicopathological factors. Int Canc Conf J.

[9] Fukuhara N, Tanino Y, Sato S (2015). Early detection of colon cancer by increased serum level of Krebs von der Lungen-6 in a patient with dermatomyositis-associated interstitial pneumonia. Sarcoidosis Vasc Difuse Lung Dis.

[10] Tsuchie H, Miyamoto S, Kataoka Y (2015). Rheumatoid arthritis with high serum KL-6 complicating malignant tumor: two case reports. Open J Orthoped.

[11] Yonenaga Y, Kurosawa M, Higashide S (2021). Gallbladder cancer detected by elevated serum KL-6 levels during the follow-up of interstitial pneumonia: a case report. Int Cancer Conf J.

[12] Guo Q, Tang W, Inagaki Y (2006). Clinical significance of subcellular localization of KL - 6mucin in primary colorectal adenocarcinoma and metastatic tissues. World J Gastroenterol.

[13] Tanaka S, Hattori N, Ishikawa N (2012). Krebs von den Lungen-6(KL-6) is a prognostic biomarker in patients with surgically resected nonsmall cell lung cancer. Int J Cancer.

[14] Tang W, Inagaki Y, Kokudo N (2005). KL-6 mucin expression in carcinoma of the ampulla of Vater: association with cancer progression. World J Gastroenterol.

[15] Gendler SJ, Spicer AP (1995). Epithelial mucin genes. Annu Rev Physiol.

[16] Pemberton LF, Rughtetti A, Taylor-Papadimitriou J (1996). The epithelial mucin MUC1 contains at least two discrete signals specifying membrane localization in cells. J Biol Chem.

[17] Ligtenberg MJ, Buijs F, Vos HL (1992). Suppression of cellular aggregation by high levels of episialin. Cancer Res.

[18] Wesseling J, van der Valk SW, Vos HL (1995). Episialin (MUC1) overexpression inhibits integrin-mediated cell adhesion to extracellular matrix components. J Cell Biol.

[19] Wesseling J, van der Valk SW, Hilkens J (1996). A mechanism for inhibition of E-cadherin-mediated cell-cell adhesion by the membrane-associated mucin episialin/MUC1. Mol Biol Cell.

[20] Kohlgraf KG, Gawron AJ, Higashi M (2003). Contribution of the MUC1 tandem repeat and cytoplasmic tail to invasive and metastatic properties of a pancreatic cancer cell line. Cancer Res.

[21] Hollingsworth MA, Swanson BJ (2004). Mucins in cancer: protection and control of the cell surface. Nat Rev Cancer.

[22] Liu X, Yi C, Wen Y (2014). Interactions between MUC1 and p120 catenin regulate dynamic features of cell adhesion, motility, and metastasis. Cancer Res.

[23] Yoshida K, Yasufuku I, Terashima M (2022). International retrospective cohort study of conversion therapy for stage IV gastric cancer 1 (CONVO-GC-1). Ann Gastroenterol Surg.

[24] Al-Batran SE, Goetze TO, Mueller DW (2017). The Renaissance (AIO-FLOT5) trial: effect of chemotherapy alone vs. chemotherapy followed by surgical resection on survival and quality of life in patients with limited-metastatic adenocarcinoma of the stomach or esophagogastric junction—a phase III trial of the German AIO/CAO-V/CAOGI. BMC Cancer.

[25] Tsuburaya A, Mizusawa J, Tanaka Y (2014). Neoadjuvant chemotherapy with S-1 and cisplatin followed by D2 gastrectomy with para-aortic lymph node dissection for gastric cancer with extensive lymph node metastasis. Br J Sur.

